# D-amino acid enhanced the sensitivity of avian pathogenic *Escherichia coli* to tetracycline and amikacin

**DOI:** 10.3389/fvets.2025.1553937

**Published:** 2025-03-19

**Authors:** Jing Wu, Bin Yang, Wei Jiang, Huifang Yin, Xiangan Han, Lili Zhang

**Affiliations:** ^1^Key Laboratory of Protection and Utilization of Biological Resources in Tarim Basin of Xinjiang Production and Construction Corps, Engineering Laboratory of Tarim Animal Diseases Diagnosis and Control, Xinjiang Production and Construction Corps, College of Life Science, Tarim University, Alar, China; ^2^Shanghai Veterinary Research Institute, The Chinese Academy of Agricultural Sciences, Shanghai, China; ^3^Engineering Research Center for the Prevention and Control of Animal Original Zoonosis, College of Life Science, Longyan University, Longyan, Fujian, China

**Keywords:** avian pathogenic *Escherichia coli*, biofilm, D-amino acid, antibiotics, susceptibility

## Abstract

Avian pathogenic *Escherichia coli* (APEC) biofilm formation has led to increased antibiotic resistance, presenting a significant challenge for the prevention and control of the disease. While certain D-amino acids (D-AAs) have been shown to inhibit the formation of various bacterial biofilms, role in APEC biofilms remains unexplored. This study investigates the effects of 19 different D-AAs on clinically isolated APEC biofilm. The results showed that D-tyrosine (D-Tyr), D-leucine (D-Leu), D-tryptophan (D-Trp), and D-methionine (D-Met) can reduce APEC formation by over 50% at a concentration of 5 mM. Subsequently, four D-AAs were selected for combination treatment with antibiotics (ceftazidime, amikacin, tetracycline, and ciprofloxacin). The findings reveal that D-Tyr enhance the sensitivity of APEC to amikacin and tetracycline, while D-Met increases the sensitivity of APEC to amikacin. The mechanisms by which D-Tyr and D-Met enhance antibiotic sensitivity were further investigated. Following treatment with D-Tyr and D-Met, scanning electron microscope (SEM) observations indicated a reduction in the number of bacteria on the surface of the cell crawl, but the shape and structure of the cells remain unchanged. Notably, the surface hydrophobicity was decreased by 33.86% and 56%, and the output of extracellular polysaccharide was decreased by 46.63% and 57.69%, respectively. Additionally, genes related to biofilm synthesis (*pgaA, pgaC*, and *luxS*) were down-regulated (*p* < 0.05), whereas porin protein-encoding genes (*ompC* and *ompF*) were up-regulated (*p* < 0.05), which inhibited formation of biofilm and enhanced the sensitivity of APEC to amikacin and tetracycline and by decreasing the hydrophobicity and extracellular polysaccharide content on cell surface and up-regulated porin genes and down-regulating the genes related to biofilm formation. According to the different D-AAs involved in this study, it can provide new ideas for the treatment of APEC.

## 1 Introduction

Avian pathogenic *Escherichia coli* (APEC) is one of the most prevalent pathogens, serving as a primary cause of mortality and morbidity within the poultry industry, resulting in significant economic losses (1). Serotypes O_1_, O_2_, and O_78_ are the predominant serotypes responsible for the epidemic of avian colibacillosis, collectively accounting for over 50% of APEC-related issues (2–5). Biofilm formation is a critical virulence factor of APEC, as it improves the survival ability of APEC in the environment, enhances resistance to the host immune system, and increases bacterial drug resistance (6). Controlling the formation of APEC biofilm is an effective strategy for preventing and managing avian colibacillosis. Recent studies have demonstrated that D-amino acids (D-AAs) play a significant role in both the inhibition of formation and dissipation of bacterial biofilms (7, 8). Kolodkin-Gal scholars found that D-Tyr may by integrating the peptidoglycan or a combination of cell wall proteins receptor TasA, caused by *Bacillus subtilis* of amyloid in the extracellular matrix reduction and decomposition, thereby inhibition of biofilm formation and decompose has formed biofilms (8). Moreover, D-Tyr can also inhibit the accumulation of matrix proteins by attaching to the cell wall of *Staphylococcus aureus* and preventing protein localization on the cell surface, thus inhibiting the formation of biofilm (9). The biofilm dispersion signal factor released by D-AAs can change the peptidoglycan composition of bacterial cell wall and regulate the cell gene expression mode, and inhibit biofilm formation by binding with bacterial proteins (10). However, the antibacterial and anti-biofilm properties of various D-AAs differ when tested against the same bacteria, and conversely, the efficacy of a specific D-AAs can vary against different bacterial strains (11, 12). For example, D-Phenylalanine (D-Phe), D-proline (D-Pro), and D-tryptophan (D-Trp) exhibited the most pronounced inhibition and dispersion effects on *Staphylococcus aureus* biofilm, while other D-AAs had little or no effect (11). D-Leu, D-Met, D-Trp, and D-Tyr had the most obvious dispersion effect on *Bacillus subtilis* biofilm, while D-alanine (D-Ala) and D-Phe did not exhibit any corresponding dispersion effects (12).

Recent advancements in D-AAs research have significantly expanded our understanding of their potential applications in antimicrobial therapy. Notably, the interaction between D-AAs and antibiotic efficacy has emerged as a promising area of investigation. Innovative approaches have been developed, including the combination of D-AAs with photothermal hydrogels for the targeted treatment of prosthetic joint infections (13). Moreover, scientific evidence demonstrates that the integration of D-AAs with conventional drugs can substantially enhance bacterial susceptibility to these therapeutic agents (14). Particularly noteworthy is the discovery of synergistic effects when D-AAs are co-administered with established antibacterial compounds, specifically tetrakis (hydroxymethyl) phosphonium sulfate (THPS) and ethylenediamine-N,N'-disuccinic acid (EDDS), resulting in markedly improved bactericidal outcomes (15). These findings collectively underscore the potential of D-AAs as valuable adjuvants in antimicrobial strategies.

This study investigates the antibacterial and anti-biofilm effects of D-AAs on APEC, as well as their potential to enhance the sensitivity of antibiotics against APEC. The broth microdilution method was used to determine the minimum inhibitory concentration (MIC) of various antibiotics, while the impact of D-AAs on APEC biofilm formation was assessed. The combinations of D-AAs with ceftazidime, tetracycline, amikacin, and ciprofloxacin were evaluated using the broth micro-checkerboard assay. Furthermore, the mechanisms by which D-AAs inhibit APEC biofilm formation were examined, which providing a foundation for the prevention and control of APEC.

## 2 Materials and methods

### 2.1 Bacterial strains and growth conditions

Clinical isolates of APEC 2309128 (O_1_), 230959 (O_1_), DE17 (O_2_), E940 (O_2_), 20170119 (O_2_), 230992 (O_78_), 2309149 (O_78_), and 230953 (O_78_; Presented by Han Xiangan, Researcher of Shanghai Veterinary Research Institute, Chinese Academy of Agricultural Sciences). Unless otherwise indicated, the bacteria were grown in Mueller-Hinton (MH) broth, Luria Bertani (LB) broth, or on a solid medium supplemented with 1.5% agar.

### 2.2 Biofilm formation of APEC

Biofilm formation was quantified using crystal violet (CV) staining (16, 17). Briefly, overnight bacterial cultures were diluted 1:100 in fresh LB broth. A 200 μL aliquot of the diluted bacterial suspension was added to each well of a 96-well plate, with eight replicates prepared for each strain. Sterile LB medium was used as a control. The plates were incubated statically at 25°C for 8, 12, 16, and 20 h. After incubation, the wells were gently washed three times with phosphate-buffered saline (PBS). The biofilms were then stained with 200 μL of 1% CV for 30 min at room temperature. Following staining, the wells were rinsed with distilled water, air-dried, and treated with 200 μL of 95% ethanol. The OD_595_ for each well was measured using a Synergy 2 microplate reader (Biotek, VT, USA). According to literature (18), the criteria for determining biofilm formation ability were as follows: Critical threshold (ODc) = mean value of negative blank control + (3 × standard deviation of negative control). OD ≤ ODc, no biofilm ability (–); ODc < OD ≤ 2 × ODc, weak biofilm forming ability (+); 2 × ODc < OD < 4 × ODc, medium biofilm forming ability (++); 4 × ODc < OD, strong biofilm forming ability (+++).

### 2.3 Biofilm inhibition of D-amino acid

The detection method was the same as 2.2. The bacteria cultured overnight were diluted in a ratio of 1:100 in fresh LB broth containing different concentrations of D-AAs (0, 0.156, 0.313, 0.625, 1.25, 2.5, 5 mM), take 200 μL of the diluted bacterial solution and add it into the 96-well plate, repeat 6 wells for each strain, sterile LB medium was used as control, static culture at 25°C for 16 h. CV staining was used to quantify the biofilm formation. To measure biofilm degradation, the absorbance of the solubilized dye was measured at 595 nm and the percentage of biofilm degradation was determined by the following equation: Biofilm degradation = [(Untreated OD_595_-Treated OD_595_)/Untreated OD_595_] × 100. Each data point was averaged from at least six replicate wells (19).

### 2.4 Determination of minimal inhibitory concentration

Minimal inhibitory concentration (MICs) were determined by a microtiter broth dilution method (96-well polystyrene plates), as recommended by the Clinical and Laboratory Standards Institute (CLSI) (20). The MIC of APEC was determined using ampicillin (AMP), ceftazidime (CAZ), cefotaxime (CTX), gentamicin (CN), amikacin (AMK), tetracycline (TE), doxycycline (DO), ciprofloxacin (CIP), and enrofloxacin (ENR) were selected. Antibiotics were purchased from Beijing Solaibao Technology Co., LTD. Briefly, a 2-fold serial dilution of antibiotics was prepared, with concentrations ranging from 256 to 0.25 μg/mL. Bacterial suspensions were adjusted to a concentration of 1 × 10^5^ CFU/mL, and the minimum inhibitory concentration (MIC) was determined using MH broth. A control containing only inoculated broth was included and incubated at 37°C for 16–20 h. The MIC endpoint was defined as the lowest antibiotic concentration at which no visible bacterial growth was observed. This was further confirmed by comparing the optical density at 600 nm (OD_600_) with the blank control, showing no statistical difference.

### 2.5 Broth micro-checkerboard assay

Broth Micro-Checkerboard was used for D-AAs and antibiotics combination as previously reported with slight modifications (21, 22). First, 100 μL of MH broth was added to columns 1–12 of the 96-well plate. Then, 100 μL of antibiotics with a concentration of 1,024 μg/mL was added to column 2. The antibiotics were serially diluted 2-fold in MH medium across columns 2–12 of the plate, the concentration of antibiotics ranges from 256 to 0.25 μg/mL. Fifty microliter of ploidy dilution D-AAs dilution add it to row A-G of the plate, the concentration of D-AAs in row A-G was 0, 0.156, 0.313, 0.625, 1.25, 2.5, and 5 mM, respectively. Except for A_12_, 2.0 × 10^5^ CFU/mL of 50 μL bacterial suspension was then added to each well. Fifty microliter fresh MH broth medium was added to A12 and H_1_, both blank (A_12_ plate) and positive (H_1_ plate) controls were set up, and the plates were incubated at 37°C for 16–20 h.

### 2.6 Determination of growth curves

To verify whether the decreased ability of bacterial biofilm formation caused by D-AAs is due to inhibited bacterial growth, a high-throughput real-time microbial growth analyzer (Tianjin Jieling Instrument Manufacturing Co., LTD.) was employed to assess the bacterial growth curve and examine the impact of D-AAs on bacterial growth. The specific procedure was as follows: overnight-cultured APEC (DE_17_) was diluted in LB broth with or without D-AAs at a ratio of 1:100. The diluted bacterial solution was then added to the test plate, with three replicates for each sample. In the high-throughput real-time microbial growth analyzer, the bacterial solution was incubated at 37°C with shaking at 180 rpm for 24 h. The OD_600_ value of the bacterial solution was measured every hour to construct the growth curve.

### 2.7 Scanning electron microscopy analysis

Avian pathogenic *Escherichia coli* cell structure and architecture of the biofilms formed in presence with the different concentration of D-AAs were analyzed by SEM (23, 24). Briefly, the bacteria cultured overnight were diluted in a ratio of 1:100 in fresh LB broth containing 5 mM of D-AAs, add to 24-well cell culture plates containing cell crawling tablets (6 × 6 mm square, Biosharp), respectively, 1 mL per well, 25°C for 16 h. APEC cells grown in D-AA-free medium were utilized as control and gently washed three times with PBS to remove non-adherent bacteria. Then adherent bacteria were fixed and dehydrated. The plates were fixed with 2.5% glutaraldehyde for 4 h at 4°C. The surfaces were washed thrice with 0.01 M PBS, and the bacteria were then dehydrated by different concentrations of ethanol (30%, 50%, 70%, 90%, 95%, and 100%) for 20 min each. After critical-point drying and coating by gold sputter, samples were examined using a scanning electron microscope (SEM; Thermo Fisher Scientific, USA).

### 2.8 Laser confocal scanning microscopy analysis

Avian pathogenic *Escherichia coli* cells were cultured as SEM analysis described, and APEC were dyed with a Live/Dead backlight bacterial viability kit with DMAO and PI (Beyotime) as previously described. Briefly, biofilm was washed three times with PBS to remove non-adherent bacteria. Then adherent bacteria were stained using the Live/Dead backlight bacterial viability kit for 15 min at 37°C in the dark. Samples were subsequently analyzed with a laser confocal scanning microscope (Nikon A1, Japan), Living cells are green and dead cells are red.

### 2.9 Cell surface hydrophobicity analysis

Cell surface hydrophobicity (CSH) was tested as previously described (23, 25). Briefly, the collected cells were washed three times through phosphate-urea-magnesium (PUM) buffer. PUM buffer as blank control, the 600 nm absorbance value of the bacterial solution was controlled in the range of 0.4–0.6 and recorded as OD_0_. The diluted bacterial suspension was added with n-hexadecane at 4:1 ratio, and then left for 15 min after vigorous shaking, take the lower water phase, measure the light absorption value at OD_600_, and record it as OD_1_. The decrease in OD value at 600 nm of the aqueous phase was taken as a measure of H%, which was calculated with the formula: H% = [(OD_0_-OD_1_)/OD_0_] × 100%. The experiment was repeated three times.

### 2.10 Production of exopolysaccharides assay

Ethanol was used for the extraction and precipitation of the EPS (23, 26, 27). Briefly, inoculated with 1% bacterial cultures (OD_600_ = 1), the LB broth with and without of D-AAs was incubated at 25°C and 180 rpm for 16 h. After incubation, bacterial suspension was centrifuged for 15 min (3,949 × g, 4°C), then the supernatant (0.22 μm) was filtered. Four volumes frozen ethanol was added to filtrate and stored at 4°C for 24 h to precipitate the EPS. The precipitated EPS were centrifuged at 16,904 × g (4°C) for 20 min, and the supernatant was discarded. The precipitation was washed twice with 95% ethanol and dried naturally at room temperature. To remove proteins, n-butyl alcohol and proteinase K were used. The aqueous layer was collected followed by dialysis with distilled water overnight. The liquid was lyophilized as an EPS sample for use.

### 2.11 Analysis of gene transcription level

To explore further the possible mechanism of D-AAs inhibiting bacterial biofilm and enhancing antibiotic sensitivity, quantitative Real-Time PCR (qRT-PCR) was performed to explore whether D-AAs affects the transcription level of APEC antibiotic sensitivity related genes. Those antibiotic resistance related genes include exopolysaccharides-encoding gene (*pgaA* and *pgaC*), autoinducer-2 synthesis gene (*luxS*), the selected efflux pump-encoding gene (*tolC*), and porin protein-encoding genes (*ompC* and *ompF*). The primers for the target genes and the internal control gene *dnaE* were shown in Table 1. Briefly, the bacteria were grown in LB at 37°C to mid-log phase (OD_600_ = 1.0), and total RNA was extracted by TRIzol reagent. The reverse transcription kit (HiScript^®^ III RT SuperMix for qPCR + gDNA wiper, Vazyme) was used to remove DNA from total RNA and reverse transcription. The qPCR experiment was performed using the SYBR green PCR mix (Vazyme, Nanjing, China). The target genes were examined three times, and relative changes in gene expression levels were assessed using the 2^−ΔΔCt^ method (28).

**Table 1:** RT-qPCR primers used in this study.

**Primer name**	**Sequence (5'−3')**	**Product size (bp)**	**Source**
RT-*dnaE*-F	ccgattgaggccatcatcga	114	This study
RT-*dnaE*-R	cagtttttccagcactcgcc		This study
RT-*ompC*-F	ggcgacacttacggttctga	92	This study
RT-*ompC*-R	ccgtcaaccagaccgaagaa		This study
RT-*ompF*-F	aaaaacgagcgtgacactgc	125	This study
RT-*ompF*-R	tcttgcaggttggtacggtc		This study
RT-*luxS*-F	gaacgtctaccagtgtggca	84	This study
RT-*luxS*-R	acgtcacgttccagaatgct		This study
RT-*pgaA*-F	atctataaactggcggggcg	148	This study
RT-*pgaA*-R	aattggcatcgtcaatcgcg		This study
RT-*pgaC*-F	ctggatgctgagtctggcaa	108	This study
RT-*pgaC*-R	cctctcagaatcccgcgatc		This study
RT-*tolC*-R	acgcctacaaacaagccgta	150	This study
RT-*tolC*-R	tataacgcgcatttgccagc		This study

### 2.12 Statistical analysis

All experimental data were recorded in Excel. Statistical analyses were conducted using R software [version 4.4.1, (47)]. An independent sample *t*-test was employed to determine whether there were significant differences between the treated groups and the control group. For experiments with three or six replicates per group, a corrected *t*-test (Welch's *t*-test) was applied. The analysis primarily utilized the *ggplot2* and *tidyverse* packages. A two-tailed *P*-value of < 0.05 was considered statistically significant.

## 3 Results

### 3.1 Biofilm formation ability of APEC

**Figure 1:**
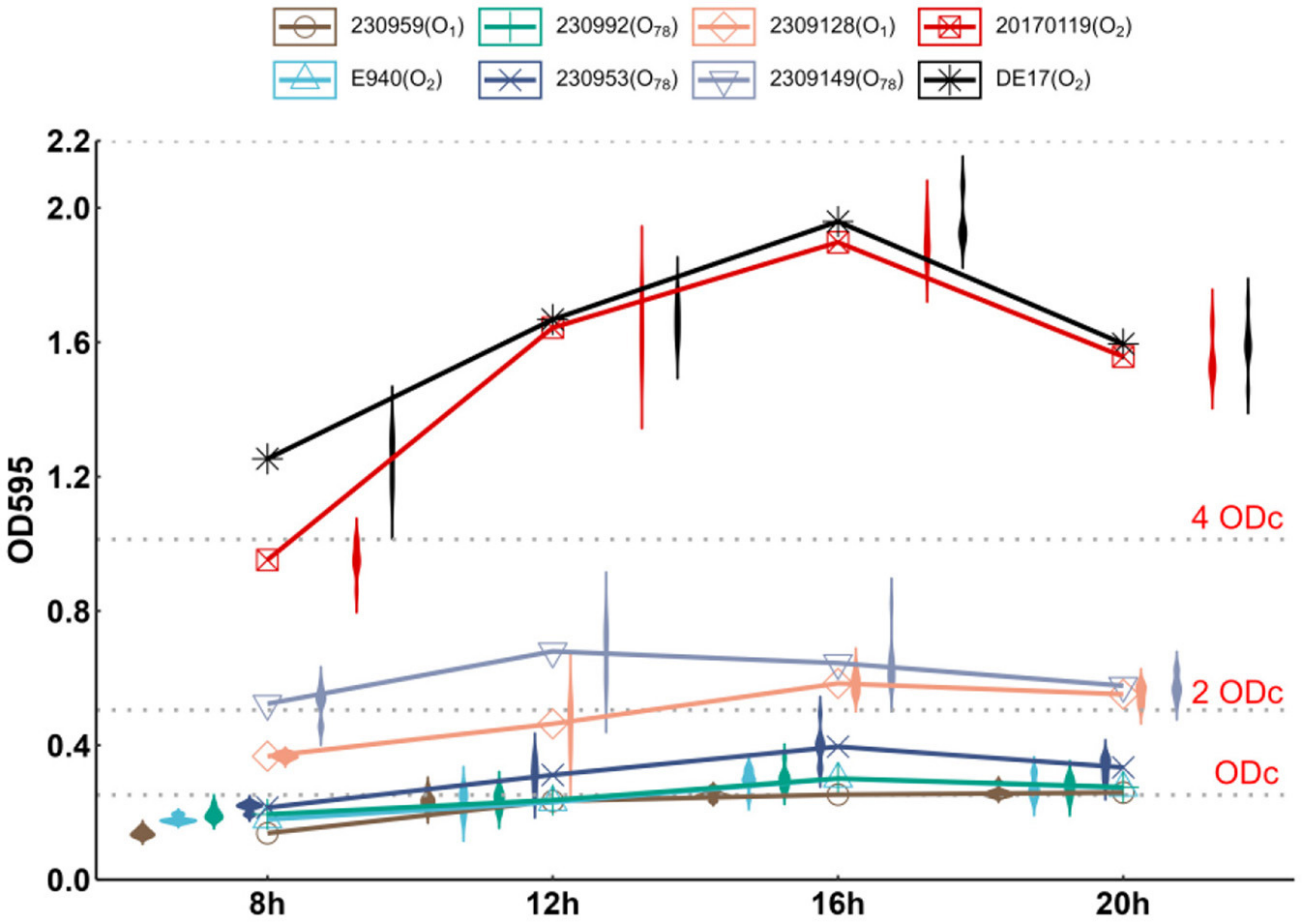
Biofilm-forming ability of APEC. The biofilm-forming ability of 7 clinically isolated APEC at 8, 12, 16, and 20 h at 25°C was quantitatively determined by crystal violet staining. 2309149 (O_78_) had the strongest biofilm formation ability at 12 h, DE17 (O_2_), 20170119 (O_2_), 2309128 (O_1_), E940 (O_2_), 230992 (O_78_), and 230953 (O_78_) had the strongest biofilm formation ability at 16 h.

With the exception of strain 2309149 (O_78_), different serotypes of APEC had the strongest ability to form biofilms at 16 h after inoculation. The results of biofilm detection of DE17 (O_2_) and 20170119 (O_2_) were OD ≥ 4 × ODc, judged as strong biofilm forming ability, the results of biofilm detection of 2309128 (O_1_) and 2309149 (O_78_) were 2 × ODc < OD < 4 × ODc, judged as medium biofilm forming ability, the results of biofilm detection of E940 (O_2_), 230992 (O_78_), and 230953 (O_78_) were ODc < OD ≤ 2 × ODc, judged as weak biofilm forming ability, the results of biofilm detection of 230959 (O_1_) was OD ≤ ODc, Judged as no biofilm forming ability (Figure 1).

### 3.2 Anti-biofilm potential of D-AAs

**Figure 2:**
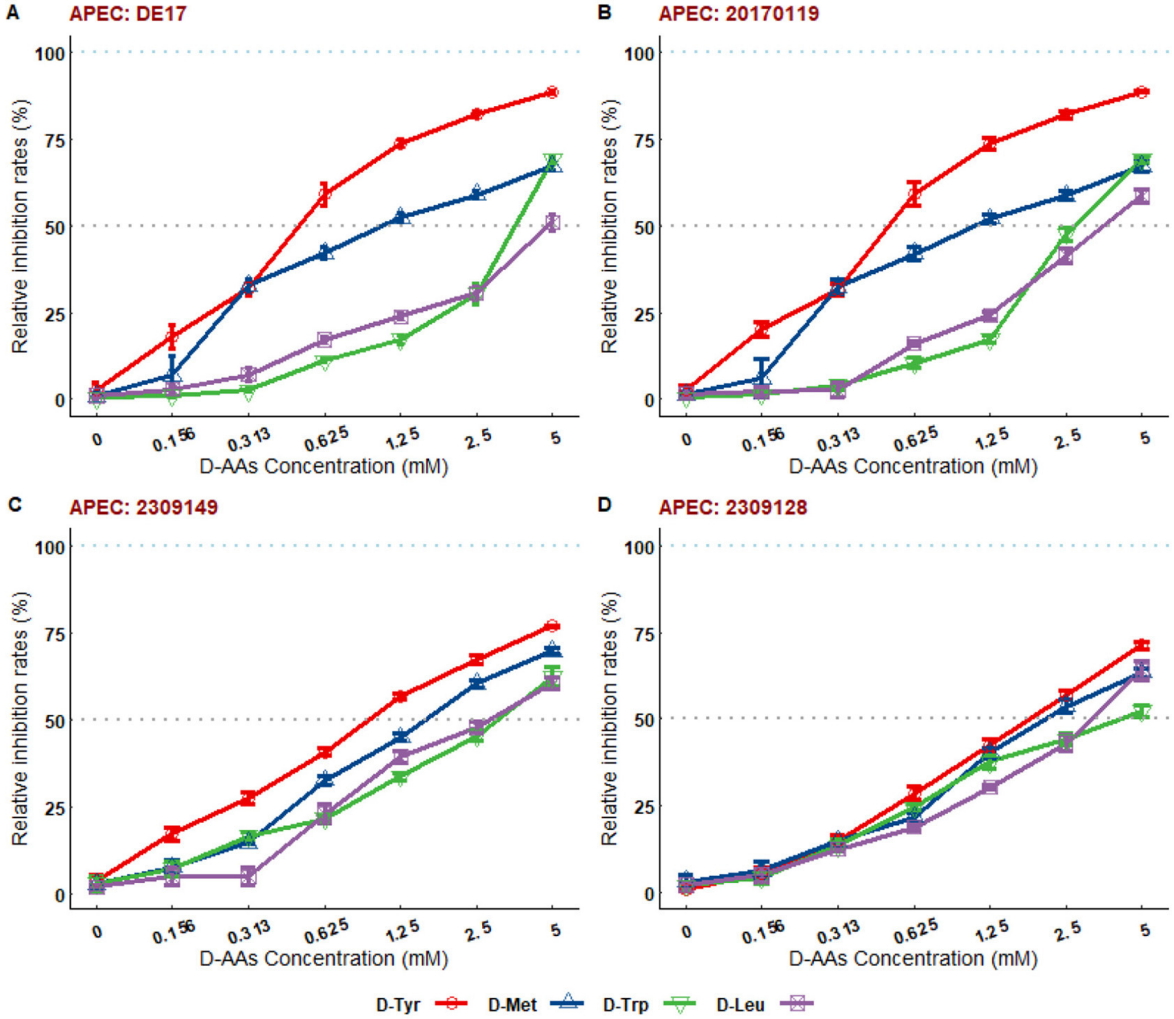
The relative inhibition rates of D-AAs on biofilms of different serotypes of APEC. **(A)** Relative inhibition rate of D-AAs on DE17 strain biofilm; **(B)** Relative inhibition rate of D-AAs on 20170119 strain biofilm; **(C)** Relative inhibition rate of D-AAs on 2309149 strain biofilm; **(D)** Relative inhibition rate of D-AAs on 2309128 strain biofilm. Mature biofilms formed over 16 h were cultured overnight in 96-well plates at 25°C, with D-AAs concentrations ranging from 0.156 to 5 mM. Biofilm formation was quantified using crystal violet staining. The biofilm degradation rate (%) was calculated as [(OD_595_ of untreated group - OD_595_ of treated group)/OD_595_ of untreated group] × 100. The results demonstrated that D-Tyr, D-Met, D-Trp, and D-Leu exhibited dose-dependent inhibitory effects on biofilms of different clinical strains. At a concentration of 5 mM, the inhibition rates of all D-AAs exceeded 50%.

Based on the results of biofilm detection, two avian pathogenic *Escherichia coli* strains (DE17 and 20170119) with strong biofilm forming ability were selected as test strains for this study, and 19 types of D-AAs were assessed for their biofilm inhibition activities. The results showed that D-AAs had dose-dependent anti-biofilm effects (Supplementary Tables 1, 2). At 5 mM, 19 kinds of D-AAs could inhibit the biofilm of two strains of APEC, among them, the inhibition rates of D-Tyr, D-Met, D-Leu, and D-trp on the biofilm of the two strains were all >50%. In order to further verify the universality of D-Tyr, D-Met, D-Leu, and D-trp on APEC biofilm inhibition, the inhibition ability of D-Tyr, D-Met, D-Leu, and D-trp on the biofilm of the strains with medium biofilm forming ability (2309128 and 2309149) was tested. The inhibition rates of D-Tyr, D-Met, D-Leu, and D-trp on 2309128 and 2309149 biofilms were all >50% (Figure 2). Four kinds of D-AAs, D-Tyr, D-Met, D-Leu, and D-trp, were used as research materials in the follow-up study.

### 3.3 D-AAs increases antibiotic sensitivity to APEC

The MIC and sensitivity of nine antibiotics were evaluated for four strains of APEC (see Supplementary Table 2). The strains 2309128 (O_1_), 20170119 (O_2_), and 2309149 (O_78_) exhibited multiple antibiotic resistances. In addition, strain 2309149 (O_78_) was moderately sensitive to fluoroquinolones (CIP, ENR), Strains 2309128 (O_1_) and 20170119 (O_2_) were resistant to β-lactam antibiotics (AMP, CAZ, and CTX), aminoglycoside antibiotics (GN, AMK), tetracycline antibiotics (TE and DO) and fluoroquinolones (ciprofloxacin, enrofloxacin) all developed resistance. Ceftazidime, amikacin, tetracycline, and ciprofloxacin were used in combination with D-AAs (D-Tyr, D-Met, D-Leu, and D-trp) according to the results of MIC determination of four strains. The results are shown in Table 2. D-Tyr enhanced the sensitivity of amikacin and tetracycline to different serotypes of APEC, and D-Met enhanced the sensitivity of amikacin to different serotypes of APEC.

**Table 2:** D-AAs combined with antibiotics against APEC *in vitro*.

	**2309128**		**DE17**		**20170119**		**2309149**	
	**ABX**	**ABX+D-AA**	**ABX**	**ABX+D-AA**	**ABX**	**ABX+D-AA**	**ABX**	**ABX+D-AA**
CAZ+D-Lue	32	32	4	4	8	8	256	256
CAZ+D-Met	32	32	4	4	8	8	256	256
CAZ+D-Trp	32	32	4	4	8	8	256	256
CAZ+D-Tyr	32	32	4	4	8	8	256	256
AK+D-Lue	128	128	8	8	64	64	64	64
AK+D-Met	128	32	8	2	32	8	64	16
AK+D-Trp	128	128	8	8	32	32	64	16
AK+D-Tyr	128	64	8	4	32	16	64	32
TE+D-Lue	256	256	4	4	32	32	32	32
TE+D-Met	256	256	4	4	32	32	32	32
TE+D-Trp	256	256	4	4	32	16	32	32
TE+D-Tyr	256	64	4	2	32	8	32	16
CIP+D-Lu	256	256	0.5	0.5	2	2	0.5	0.5
CIP+D-Met	256	256	0.5	0.5	2	2	0.5	0.5
CIP+D-Trp	256	256	0.5	0.5	2	2	0.5	0.5
CIP+D-Tyr	256	256	0.5	0.5	2	2	0.5	0.5

ABX, antibiotics; Unit, μg/mL.

In order to explore the possible mechanism of D-Tyr and D-Met enhancing drug sensitivity, the laboratory model strain DE17 was selected as the research object for follow-up experiments.

### 3.4 D-AAs had no inhibitory effect on DE17 growth

**Figure 3:**
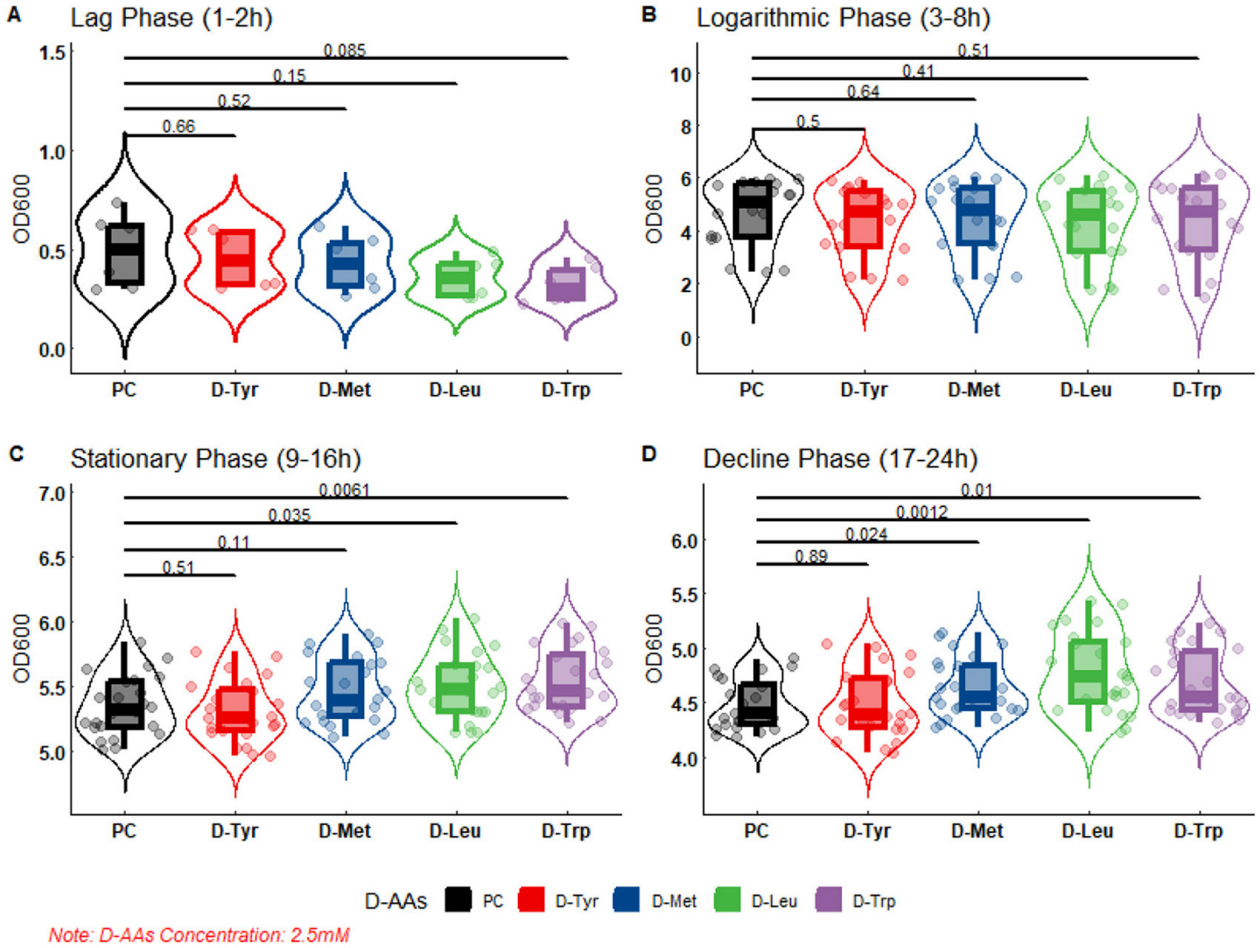
The effect of 2.5 mM D-AAs on the growth of APEC. PC, Positive Control, no added to D-AAs control group. APEC DE17 incubated at 37°C with shaking at 180 rpm for 24 h. The OD_600_ values of the bacterial cultures were measured every hour, with three replicates for each sample. The bacterial growth curve was divided into four phases: **(A)** Lag phase (1–2 h), **(B)** Logarithmic phase (3–8 h), **(C)** Stationary Phase (9–16 h), and **(D)** Decline phase (17–24 h). A-test was performed to compare the OD_600_ values of each D-AAs treatment group to the PC group. The *p*-values from the *t*-test are indicated on the horizontal lines in the figure.

**Figure 4:**
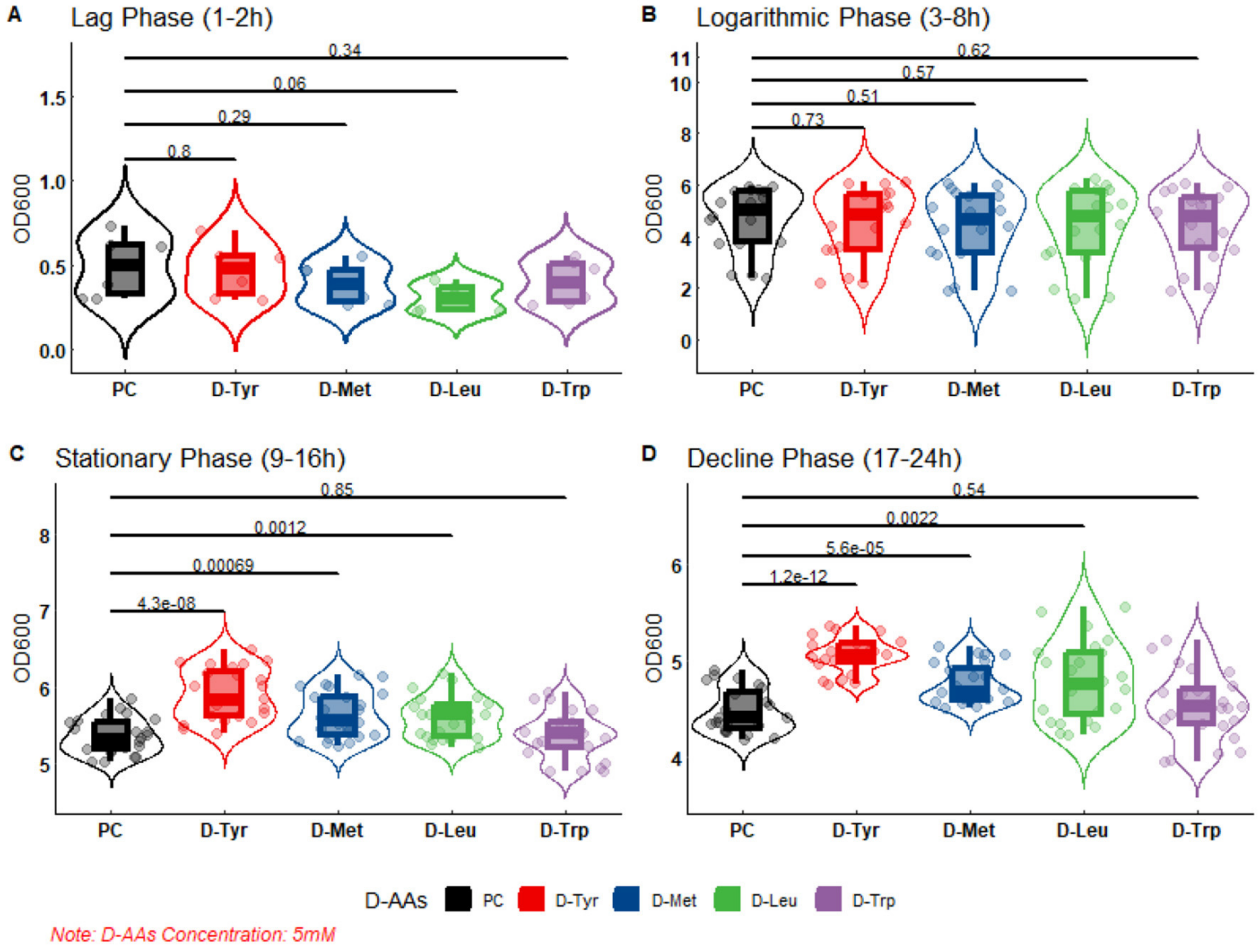
The effect of 5 mM D-AAs on the growth of APEC. PC, Positive Control, no added to D-AAs control group. APEC DE17 incubated at 37°C with shaking at 180,rpm for 24 h. The OD_600_ values of the bacterial cultures were measured every hour, with three replicates for each sample. The bacterial growth curve was divided into four phases: **(A)** Lag phase (1–2 h), **(B)** Logarithmic phase (3–8 h), **(C)** Stationary phase (9–16 h), and **(D)** Decline phase (17-24 h). A t-test was performed to compare the OD_600_ values of each D-AAs treatment group to the PC group. The *p*-values from the *t*-test are indicated on the horizontal lines in the figure.

The results of bacterial growth curve showed that the D-AAs content had no inhibitory effect on bacterial growth when it was 2.5 or 5 mM (Figures 3, 4).

As shown in the figure, at 2.5 or 5 mM, there was no difference in DE17 growth between the D-AAs treated group and the untreated group at the slow stage and logarithmic stage, and there were differences between the amino acid treated group and the untreated group at the stable stage and the decline stage. At 2.5 mM, D-Leu and D-trp significantly accelerated APEC growth (*P* < 0.05; Figure 3), and at 5 mM, D-Tyr, D-Leu, and D-Met significantly accelerated APEC growth (*P* < 0.01; Figure 4). Indicating that biofilm dispersive activity was not associated with growth inhibition.

### 3.5 D-AAs had no effect on the integrity of cell walls and membranes

**Figure 5:**
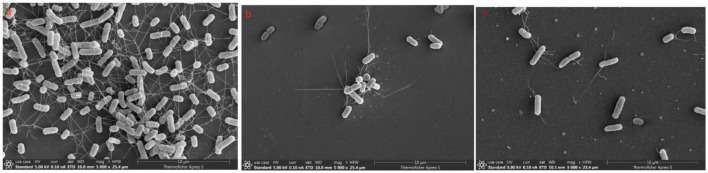
SEM of biofilms formed by APEC under different treatment conditions. **(A)** Control (without D-AAs), **(B)** D-Tyr, **(C)** D-Met. **(A)** Bacteria exhibit a smooth surface and are present in large quantities. **(B)** Bacteria maintain a smooth surface and adherence, but their quantity is significantly reduced. **(C)** Bacteria also display a smooth surface, show significantly reduced adherence, and their quantity is greatly diminished.

The SEM results showed that the number of adherent bacteria in samples treated with D-AAs was significantly reduced compared to the control group. Furthermore, changes in the surface of APEC in the D-AAs treated samples were observed, however, the cells remained intact (Figure 5).

**Figure 6:**
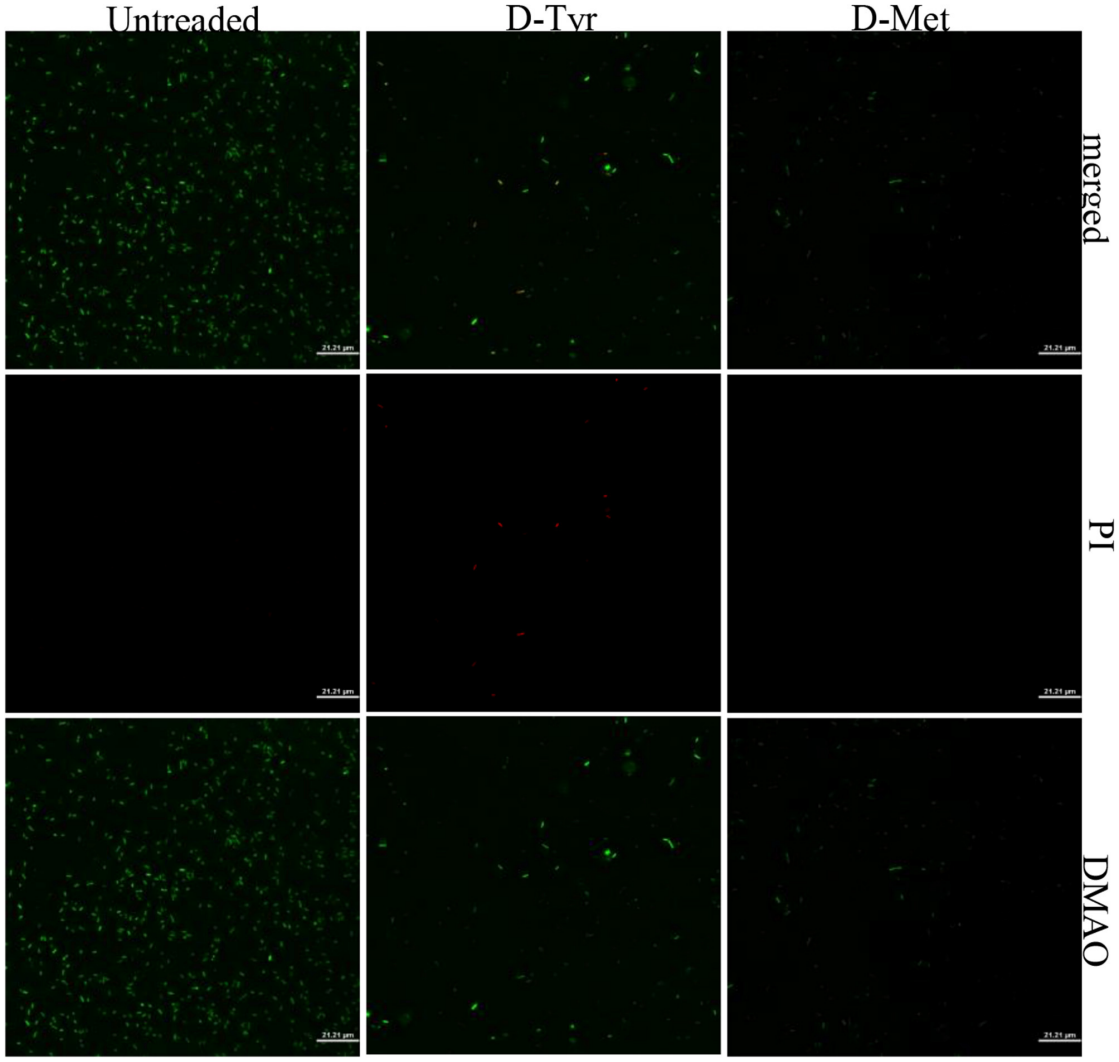
CLSM analysis of bacterial viability in a biofilm on a coverslip surface. APEC biofilms were subjected to dual fluorescence staining using the Live/Dead BacLight bacterial viability kit. Specifically, DMAO (3,6-diamino-9-methylacridinium) was employed to stain metabolically active cells, emitting green fluorescence, while propidium iodide (PI) was used to stain membrane-compromised cells, emitting red fluorescence. Analysis revealed a significant reduction in bacterial adhesion in samples treated with D-Tyr and D-Met compared to control groups, demonstrating the inhibitory effects of these D-AAs on biofilm formation and bacterial viability.

After staining with the Live/Dead backlight bacterial viability kit, CLSM results showed that compared with untreaded group, the number of attached bacteria in the samples treated with D-Tyr (5 mM) was significantly reduced, Moreover, red fluorescence appeared, and the number of attached bacteria in D-Met (5 mM) treated samples also decreased significantly, there was no red fluorescence (Figure 6).

### 3.6 D-AAs decreased cell surface hydrophobicity in APEC

**Figure 7:**
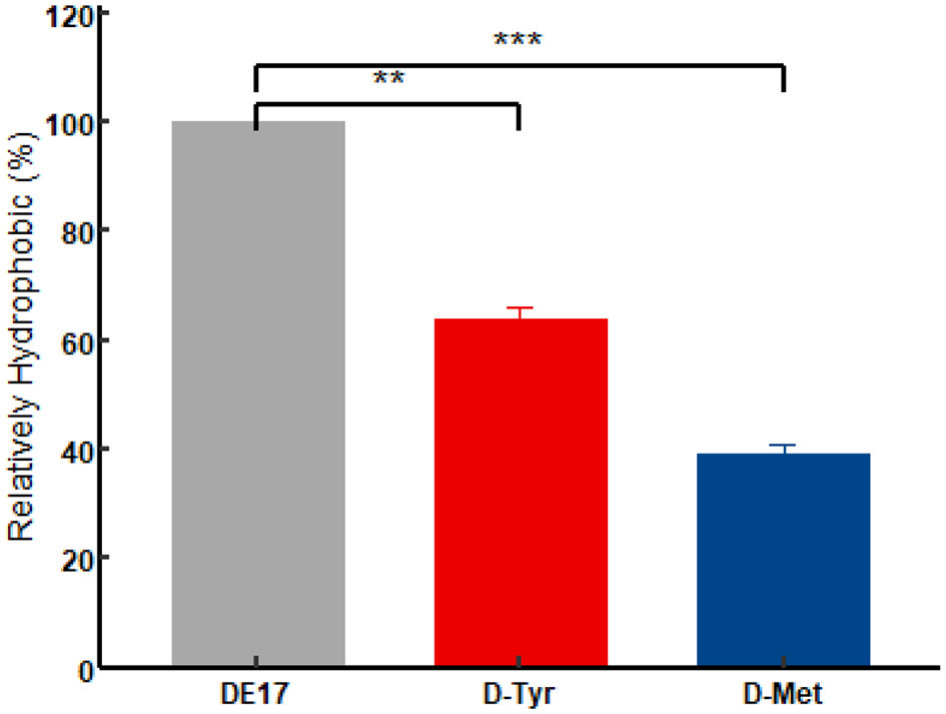
Effect of D-amino acids on the CSH of APEC. The experiment was performed in triplicate. A *t*-test was conducted to compare the H% values of each D-AAs treatment group to the control group. The D-AAs treated group compare to the untreated group was very significant decreased (^**^P < 0.01, ^***^P < 0.001).

Cell surface hydrophobicity (CSH) was positively correlated with the adhesion of cell (29). At 5 mM, D-Tyr and D-Met significantly reduced CSH (*p* < 0.01), decreased by 33.86% and 56%, respectively, thus reducing the adhesion of APEC (Figure 7).

### 3.7 D-AAs reduced the production of EPS in DE17

**Figure 8:**
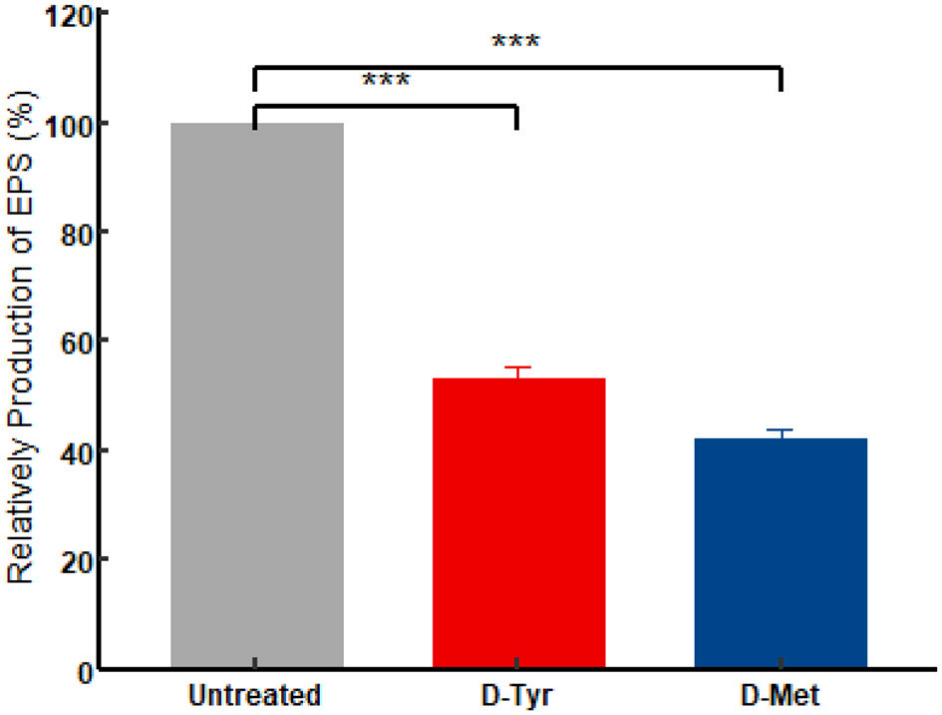
Effect of D-AAs on the EPS production of DE17. The experiment was conducted in triplicate. A *t*-test was performed to compare the relative production of extracellular polymeric substances (EPS) in each D-AAs treatment group to the control group. The significance levels of the *p*-values from the *t*-test are indicated on the horizontal lines in the figure as ^***^P < 0.001.

As shown in Figure 8, in the presence of 5 mM D-AAs (D-Tyr or D-Met), the EPS production in APEC was significantly reduced (*P* < 0.001; Figure 8). After treatment with D-Tyr, D-Met the EPS production of APEC decreased by 46.63%, 57.69%, respectively.

### 3.8 D-AAs regulates multiple biofilm related genes to inhibit biofilm formation

**Figure 9:**
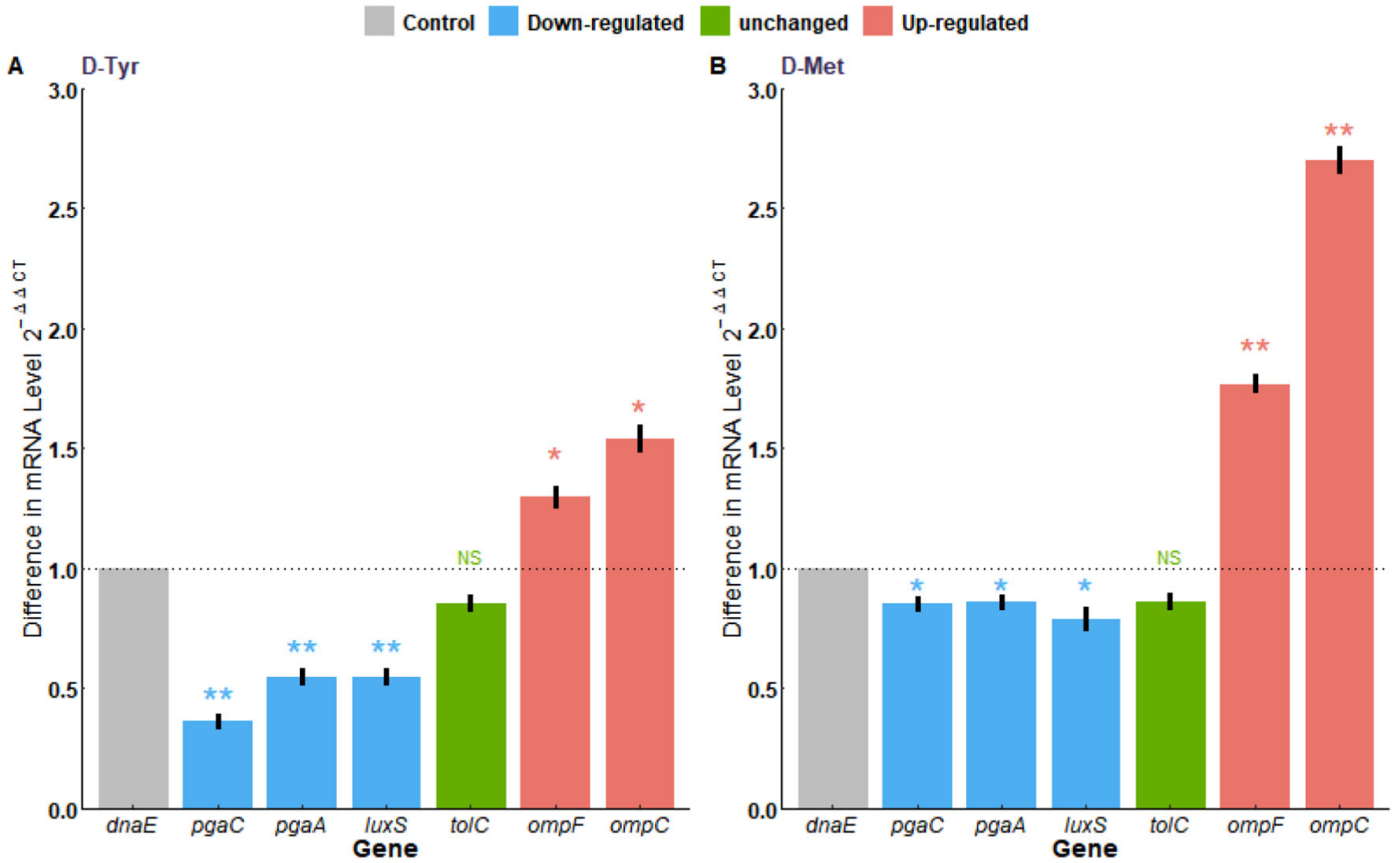
Transcriptional analysis of antibiotic resistance-related genes. **(A)** With or without the treatment of D-Try, **(B)** with or without the treatment of D-Met. Expression of *dnaE* was used as a housekeeping control. The data are representative of results from three independent experiments. The asterisks represent statistical significance, ns, not significant, *p* > 0.05; *p < 0.05, **p < 0.01.

The qPCR results showed that, the D-Tyr treated group was compared with the untreated group, the mRNA transcription levels of *ompC* and *ompF* genes were significantly up-regulated (*p* < 0.05), while the mRNA transcription levels of *luxS, pgaA*, and *pgaC* genes were significantly down-regulated (*p* < 0.01), and *tolC* mRNA levels were not statistically significant (*p* > 0.05; Figure 9A). The D-Met treated group was compared with the untreated group, The mRNA transcription levels of *ompC* and *ompF* genes were significantly up-regulated (*p* < 0.01). The mRNA transcription of *luxS, pgaA*, and *pgaC* were significantly down-regulated (*p* < 0.05), while *tolC* mRNA levels were not significantly different (*p* > 0.05; Figure 9B).

## 4 Discussion

The formation of APEC biofilm is the primary cause of chronic, persistent, and recurrent infections, as well as antibiotic resistance (6). Controlling APEC biofilm formation is a crucial target to decrease the potential risk of infection by this bacterium in poultry (30). Recent studies have shown that the D-AAs can inhibit and disperse biofilms formed by a diverse range of bacterial species, including *Bacillus subtilis, Staphylococcus aureus* and *Pseudomonas aeruginosa* (8, 31, 32). However, various D-AAs have different antibacterial and anti-biofilm properties to the same bacteria, and the same D-AAs may have distinct antibacterial and anti-biofilm properties against different bacterial species (11, 12). Consequently, this study investigates the inhibitory effects of 19 D-AAs on three serotypes of APEC biofilms were investigated in this study. This study shows that D-AAs has a dose-dependent inhibitory effect on APEC biofilm (Supplementary Tables 1, 2), in which D-Tyr, D-Met, D-Leu, and D-trp have strong inhibitory effects on various serotypes of APEC biofilms (Figure 2). The results are consistent with previous studies on *Bacillus subtilis* (12). This study found that D-AAs did not inhibit the growth of APEC *E. coli* at concentrations of 2.5 and 5 mM. However, at 2.5 mM, the levels of D-Leu and D-Trp significantly increased during the stationary and lag phases (*P* < 0.05). At 5 mM, the levels of D-Tyr, D-Met, and D-Leu significantly increased during the stationary and lag phases (*P* < 0.01). These findings are consistent with Rumbot's discovery that D-arginine, D-glutamine, and D-alanine can induce the growth of *Pseudomonas aeruginosa* (33).

Given the importance of biofilms in disease and the resistance of conventional antibiotics to APEC. In this study, D-AAs (D-Tyr, D-Met, D-Leu, and D-trp), which has obvious inhibitory effect on biofilms, was used in combination with antibiotics (CAZ, AK, TE, and CIP) to analyze whether D-AAs can improve the sensitivity of antibiotics to APEC. D-Tyr enhanced the sensitivity of amikacin and tetracycline to different serotypes of APEC, and D-Met enhanced the sensitivity of amikacin to different serotypes of APEC (Table 2). Currently, it is widely believed that D-AAs disperse biofilms, disrupting the protective effect of biofilms on the bacteria within. This forces the bacteria to transition from a biofilm state to a free-living state, making them more susceptible to being killed by bactericides, thereby enhancing the sterilization rate (10, 34).

Enhancing the uptake of antibiotics and reducing the efflux of antibiotics are beneficial measures to enhance antibiotic sensitivity. The outer membrane porin of gram-negative bacteria is a transmembrane protein that allows the passive transport of various compounds (such as antibiotics) into bacterial cells (35). When the expression of porin is down-regulated, the amount of antibiotic entering the cell will be reduced (36). OmpF and OmpC are non-specific outer membrane porins protein, and the OmpF and OmpC found in *E. coli* are trimeric β-barrel structures, through which different kinds of antibiotics can pass (37). Studies have shown that *ompF*-deficient mutants were resistant to several antibiotics, which indicates that OmpF was the main pathway for antibiotics to penetrate the outer membrane (38). It has been reported that OmpC and OmpF porins contribute to the translocation of antibiotics in the bacterial outer membrane and promote the entry of kanamycin into *E. coli* (39). Active efflux is facilitated by transmembrane efflux pumps, which export antibiotics to bacterial cells to reduce their intracellular concentration (40). AcrAB–TolC is one of the most important efflux pumps in enterobacteria, capable of squeezing a wide variety of structurally diverse compounds, including many antibiotics, from bacterial cells, thus reducing their intracellular concentrations (41). In this study, D-Tyr and D-Met can significantly enhance the transcription levels of *ompF* and *ompC* genes, but have no effect on *tolC* transcription levels. The results showed that D-Tyr and D-Met could enhance the transcription level of *ompF* and *ompC*, increase the content of intracellular antibiotics, and improve the sensitivity of antibiotics.

The biofilm is the main reason why bacteria develop drug resistance and evade the host immune mechanism. CSH is closely related to the formation of bacterial biofilm, it enhances the adhesion and agglutination of microbial cells and promotes the expansion of interfacial microcolonies (42, 43). D-AAs can inhibit initial adhesion of bacteria by reducing hydrogen bonding, changing surface potential, and hydrophilicity (44). EPS are the main components of bacterial biofilms (45). Related studies have reported that *E. coli* EPS is essential for enhancing its adhesion and drug resistance, as well as promoting the development of the host immune system (46). *pgaA* and *pgaC* are the genes encoding EPS, RT-PCR results showed that D-AAs reduced the content of bacterial exopolycans by down-regulating *pgaA* and *pgaC*, thus reducing the biofilm of bacteria. The observed enhancement in antibiotic susceptibility in this study may be attributed to the ability of D-AAs to reduce CSH and EPS production, thereby facilitating the penetration and efficacy of antibiotics.

The findings of this study hold significant implications for the prevention and control of APEC infections in the poultry industry. The ability of D-AAs to inhibit biofilm formation and enhance antibiotic sensitivity offers a promising strategy for combating APEC-related diseases. Furthermore, the combination of D-AA with antibiotics may provide a synergistic approach to overcoming antibiotic resistance, which is a major challenge in the management of APEC infections. Although this study provides valuable insights into the antibiofilm and antibiotic-enhancing effects of D-AA, it is important to acknowledge some limitations. Firstly, the research was conducted *in vitro*, and further studies are needed to evaluate the efficacy of D-AA *in vivo*. Secondly, the mechanisms by which D-AA regulates gene expression and biofilm formation require further investigation. Future research should explore the specific interactions between D-AA and bacterial proteins or receptors involved in biofilm regulation.

## 5 Conclusion

**Figure 10:**
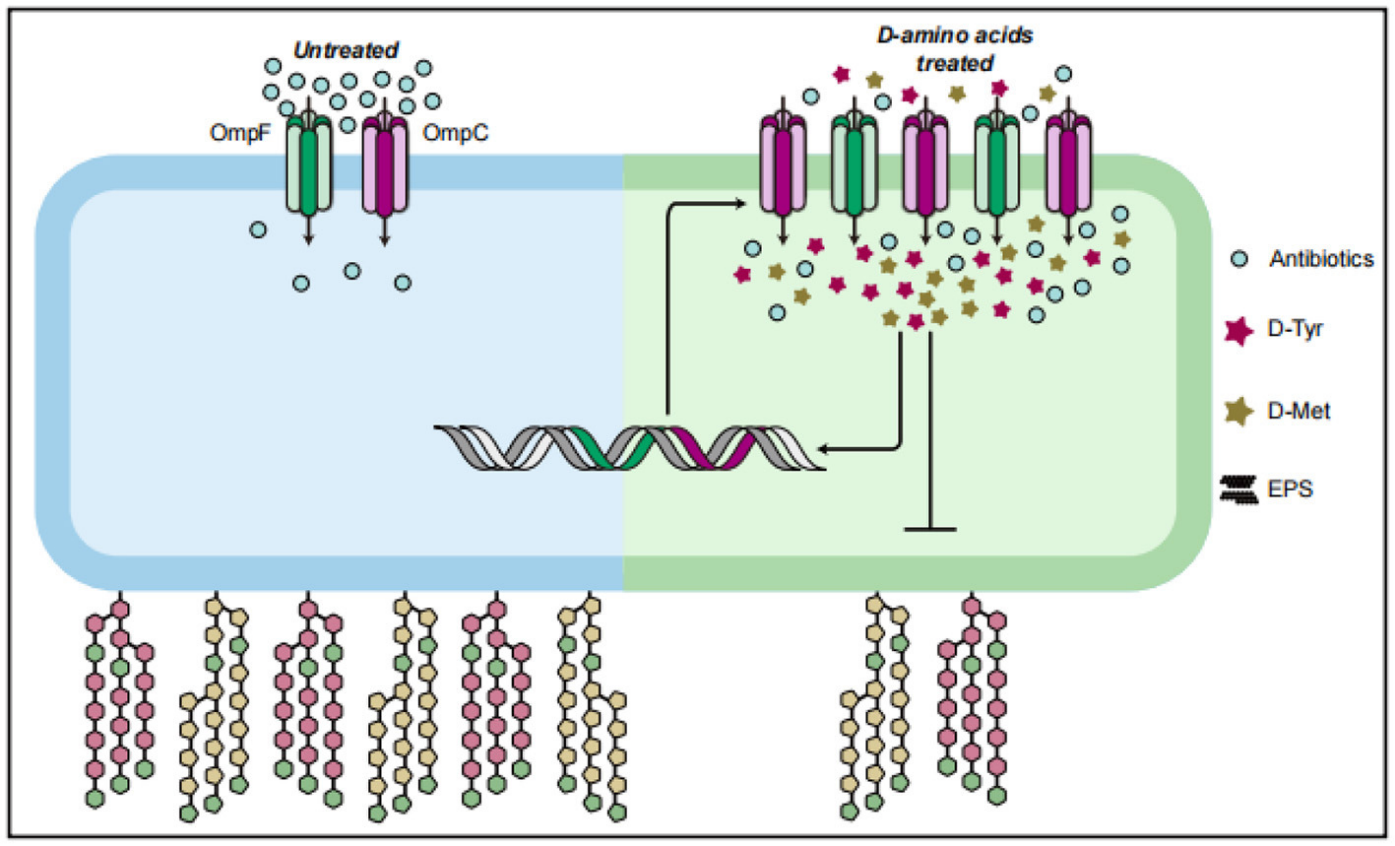
D-AAs enhance the sensitivity of APEC to antibiotics by altering its uptake of antibiotics.

AAs inhibits bacterial biofilm formation by reducing cell hydrophobicity and extracellular polysaccharide content. D-AAs up-regulates the transcription level of porin genes (*ompF* and *ompC*), down-regulates the encoding biofilm genes (*pgaA* and *pgaC*). This modulation increases the influx of antibiotics into the cells, and enhances their sensitivity to these antimicrobial agents (Figure 10). These findings highlight the potential of D-AAs as a novel strategy for controlling APEC infections and overcoming antibiotic resistance in the poultry industry. Further research is needed to explore the practical applications of D-AAs *in vivo* and to elucidate the molecular mechanisms underlying its activity.

## Data Availability

The original contributions presented in the study are included in the article/Supplementary material, further inquiries can be directed to the corresponding authors.

## References

[1] Ali A Kolenda R Khan MM Weinreich J Li G Wieler LH. Novel avian pathogenic *Escherichia coli* genes responsible for adhesion to chicken and human cell lines. Appl Environ Microbiol. (2020) 86:01068-20. 10.1128/AEM.01068-20 32769194 PMC7531953

[2] Dho-Moulin M Fairbrother JM. Avian pathogenic *Escherichia coli* (APEC). Vet Res. (1999) 30:299–316. 10367360

[3] Ewers C Janssen T Kiessling S Philipp HC Wieler LH. Molecular epidemiology of avian pathogenic *Escherichia coli* (APEC) isolated from colisepticemia in poultry. Vet Microbiol. (2004) 104:91–101. 10.1016/j.vetmic.2004.09.008 15530743

[4] Koutsianos D Athanasiou LV Mossialos D Franzo G Cecchinato M Koutoulis KC. Investigation of serotype prevalence of *Escherichia coli* strains isolated from layer poultry in Greece and interactions with other infectious agents. Vet Sci. (2022) 9:1–14. 10.3390/vetsci9040152 35448650 PMC9025756

[5] Smith KR Bumunang EW Schlechte J Waldner M Anany H Walker M. The isolation and characterization of bacteriophages infecting avian pathogenic *Escherichia coli* O1, O2 and O78 strains. Viruses. (2023) 15:1–22. 10.3390/v15102095 37896873 PMC10612097

[6] Ruan X Deng X Tan M Wang Y Hu J Sun Y. Effect of resveratrol on the biofilm formation and physiological properties of avian pathogenic *Escherichia coli*. J Proteomics. (2021) 249:104357. 10.1016/j.jprot.2021.104357 34450330

[7] Su X Cheng X Wang Y Luo J. Effect of different D-amino acids on biofilm formation of mixed microorganisms. Water Sci Technol. (2022) 85:116–24. 10.2166/wst.2021.623 35050870

[8] Kolodkin-Gal I Romero D Cao S Clardy J Kolter R Losick R. D-amino acids trigger biofilm disassembly. Science. (2010) 328:627–9. 10.1126/science.1188628 20431016 PMC2921573

[9] Hochbaum AI Kolodkin-Gal I Foulston L Kolter R Aizenberg J Losick R. Inhibitory effects of D-amino acids on *Staphylococcus aureus* biofilm development. J Bacteriol. (2011) 193:5616–22. 10.1128/JB.05534-11 21856845 PMC3187230

[10] Congmin X Haoran G Wensheng Z Xing Y Yueqing C Wenyuan W. Study on the behavior and mechanism of D-amino acid dispersing biofilm. Mater Rep. (2023) 37:1–7. 10.11896/cldb.21050076

[11] Boles BR Horswill AR. *Staphylococcal* biofilm disassembly. Trends Microbiol. (2011) 19:449–55. 10.1016/j.tim.2011.06.004 21784640 PMC3164736

[12] Lam H Oh DC Cava F Takacs CN Clardy J de Pedro MA. D-amino acids govern stationary phase cell wall remodeling in bacteria. Science. (2009) 325:1552–5. 10.1126/science.1178123 19762646 PMC2759711

[13] Milbrandt NB Tsai YH Cui K Ngompe Massado CS. Combination d-amino acid and photothermal hydrogel for the treatment of prosthetic joint infections. ACS Appl Bio Mater. (2023) 6:1231–41. 10.1021/acsabm.2c01083 36867723

[14] She P Chen L Liu H Zou Y Luo Z Koronfel A. The effects of D-Tyrosine combined with amikacin on the biofilms of *Pseudomonas aeruginosa*. Microb Pathog. (2015) 86:38–44. 10.1016/j.micpath.2015.07.009 26188263

[15] Xu D Wen J Fu W Gu T Raad I. D-amino acids for the enhancement of a binary biocide cocktail consisting of THPS and EDDS against an SRB biofilm. World J Microbiol Biotechnol. (2012) 28:1641–6. 10.1007/s11274-011-0970-5 22805946

[16] Long J Yang C Liu J Ma C Jiao M Hu H. Tannic acid inhibits *Escherichia coli* biofilm formation and underlying molecular mechanisms: biofilm regulator CsgD. Biomed Pharmacother. (2024) 175:116716. 10.1016/j.biopha.2024.116716 38735084

[17] Hu J Lv X Niu X Yu F Zuo J Bao Y. Effect of nutritional and environmental conditions on biofilm formation of avian pathogenic *Escherichia coli*. J Appl Microbiol. (2022) 132:4236–51. 10.1111/jam.15543 35343028

[18] Yu Huaying LQ. Screening biofilm and determination growth biofilm of curve in *Escherichia coli*. Chin Agric Sci Bull. (2014) 30:23–7. 10.11924/j.issn.1000-6850.2013-1796

[19] Pourhajibagher M Hosseini N Bahador A. Antimicrobial activity of D-amino acid in combination with photo-sonoactivated hypericin nanoparticles against *Acinetobacter baumannii*. BMC Microbiol. (2023) 23:23. 10.1186/s12866-023-02758-4 36658487 PMC9850556

[20] CLSI. Performance Standards for Antimicrobial Susceptibility Testing, 32th ed. CLSl Supplement M100. Wayne, PA: Clinical and Laboratory Standards Institute (2022).

[21] Rogers SA Huigens RW III Cavanagh J Melander C. Synergistic effects between conventional antibiotics and 2-aminoimidazole-derived antibiofilm agents. Antimicrob Agents Chemother. (2010) 54:2112–8. 10.1128/AAC.01418-09 20211901 PMC2863642

[22] Yan Y Yang G Li Y Mao J Wang S Zhang N. Factorial design and post-antibiotic sub-MIC effects of linezolid combined with fosfomycin against vancomycin-resistant *Enterococci*. Ann Transl Med. (2022) 10:148. 10.21037/atm-21-4595 35284561 PMC8904999

[23] Mu YQ Xie TT Zeng H Chen W Wan CX Zhang LL. Streptomyces-derived actinomycin D inhibits biofilm formation via downregulating ica locus and decreasing production of PIA in *Staphylococcus epidermidis. J Appl Microbiol*. (2020) 128:1201–7. 10.1111/jam.14543 31808241

[24] Ren Q Luo W Chi H Zhang L Chen W. Down-regulation of beta-lactam antibiotics resistance and biofilm formation by *Staphylococcus epidermidis* is associated with isookanin. Front Cell Infect Microbiol. (2023) 13:1139796. 10.3389/fcimb.2023.1139796 37234778 PMC10206261

[25] Drumm B Neumann AW Policova Z Sherman PM. Bacterial cell surface hydrophobicity properties in the mediation of *in vitro* adhesion by the rabbit enteric pathogen *Escherichia coli* strain RDEC-1. J Clin Invest. (1989) 84:1588–94. 10.1172/JCI114336 2572606 PMC304025

[26] Pei ZJ Li C Dai W Lou Z Sun X Wang H. The anti-biofilm activity and mechanism of apigenin-7-O-glucoside against *Staphylococcus aureus* and *Escherichia coli*. Infect Drug Resist. (2023) 16:2129–40. 10.2147/IDR.S387157 37070126 PMC10105580

[27] Song YJ Yu HH Kim YJ Lee NK Paik HD. Anti-biofilm activity of grapefruit seed extract against *Staphylococcus aureus* and *Escherichia coli*. J Microbiol Biotechnol. (2019) 29:1177–83. 10.4014/jmb.1905.05022 31370119

[28] Hu J Gu Y Lu H Raheem MA Yu F Niu X. Identification of novel biofilm genes in avian pathogenic *Escherichia coli* by Tn5 transposon mutant library. World J Microbiol Biotechnol. (2022) 38:130. 10.1007/s11274-022-03314-4 35688968

[29] Mirzabekyan S Harutyunyan N Manvelyan A Malkhasyan L Balayan M Miralimova S. Fish probiotics: cell surface properties of fish intestinal *Lactobacilli* and *Escherichia coli*. Microorganisms. (2023) 11:2–15. 10.3390/microorganisms11030595 36985169 PMC10052099

[30] Yin L Li Q Xue M Wang Z Tu J Song X. The role of the phoP transcriptional regulator on biofilm formation of avian pathogenic *Escherichia coli*. Avian Pathol. (2019) 48:362–70. 10.1080/03079457.2019.1605147 30958690

[31] Radkov AD Moe LA. Bacterial synthesis of D-amino acids. Appl Microbiol Biotechnol. (2014) 98:5363–74. 10.1007/s00253-014-5726-3 24752840

[32] Ercal N Luo X Matthews RH Armstrong DW. *In vitro* study of the metabolic effects of D-amino acids. Chirality. (1996) 8:24–9. 10.1002/(SICI)1520-636X(1996)8:1<24::AID-CHIR6>3.0.CO;2-G 8845278

[33] Rumbo C Vallejo JA Cabral MP Martínez-Guitián M Pérez A Beceiro A. Assessment of antivirulence activity of several D-amino acids against *Acinetobacter baumannii* and *Pseudomonas aeruginosa*. J Antimicrob Chemother. (2016) 71:3473–81. 10.1093/jac/dkw342 27605598

[34] Yang J Ran Y Liu S Ren C Lou Y Ju P. Synergistic D-amino acids based antimicrobial cocktails formulated via high-through put screening and machine learning. Adv Sci. (2024) 11:1–13. 10.1002/advs.202307173 38126652 PMC10916672

[35] Darby EM Trampari E Siasat P Solsona Gaya M Alav I Webber MA. Molecular mechanisms of antibiotic resistance revisited. Nat Rev Microbiol. (2023) 21:280–95. 10.1038/s41579-022-00820-y 36411397

[36] Fernandez L Hancock RE. Adaptive and mutational resistance: role of porins and efflux pumps in drug resistance. Clin Microbiol Rev. (2012) 25:661–81. 10.1128/CMR.00043-12 23034325 PMC3485749

[37] Nikaido H. Molecular basis of bacterial outer membrane permeability revisited. Microbiol Mol Biol Rev. (2003) 67:593–656. 10.1128/MMBR.67.4.593-656.2003 14665678 PMC309051

[38] Beeston AL Surette MG. pfs-dependent regulation of autoinducer 2 production in *Salmonella enterica* serovar Typhimurium. J Bacteriol. (2002) 184:3450–6. 10.1128/JB.184.13.3450-3456.2002 12057938 PMC135139

[39] Bafna JA Sans-Serramitjana E Acosta-Gutiérrez S Bodrenko IV Hörömpöli D Berscheid A. Kanamycin Uptake into *Escherichia coli* is facilitated by OmpF and OmpC porin channels located in the outer membrane. ACS Infect Dis. (2020) 6:1855–65. 10.1021/acsinfecdis.0c00102 32369342

[40] Webber MA Piddock LJ. The importance of efflux pumps in bacterial antibiotic resistance. J Antimicrob Chemother. (2003) 51:9–11. 10.1093/jac/dkg050 12493781

[41] Jang S. AcrAB–TolC, a major efflux pump in Gram negative bacteria: toward understanding its operation mechanism. BMB Rep. (2023) 56:326–34. 10.5483/BMBRep.2023-0070 37254571 PMC10315565

[42] Krasowska A Sigler K. How microorganisms use hydrophobicity and what does this mean for human needs? Front Cell Infect Microbiol. (2014) 4:112. 10.3389/fcimb.2014.00112 25191645 PMC4137226

[43] Youhong H. Research progress on hydrophobicity and adhesion of microbial cell surface. Int J Dermatol Venereol. (1995) 21:83–6.

[44] Si X Quan X Li Q Wu Y. Effects of D-amino acids and norspermidine on the disassembly of large, old-aged microbial aggregates. Water Res. (2014) 54:247–53. 10.1016/j.watres.2014.02.007 24576700

[45] Gheorghita AA Wozniak DJ Parsek MR Howell PL. *Pseudomonas aeruginosa* biofilm exopolysaccharides: assembly, function, and degradation. FEMS Microbiol Rev. (2023) 47:1–31. 10.1093/femsre/fuad060 37884397 PMC10644985

[46] Hufnagel DA Depas WH Chapman MR. The biology of the *Escherichia coli* extracellular matrix. Microbiol Spectr. (2015) 3:1–24. 10.1128/microbiolspec.MB-0014-2014 26185090 PMC4507285

[47] R Core Team. R: A Language and Environment for Statistical Computing. Vienna: R Foundation for Statistical Computing (2024).

