# Supplementary material for: D-amino acid enhanced the sensitivity of avian pathogenic Escherichia coli to tetracycline and amikacin
Source: Front Vet Sci. 2025 Mar 19;12:1553937. doi: 10.3389/fvets.2025.1553937 (PMC11962725; doi:10.3389/fvets.2025.1553937)
Supplement: Supplementary file 1 [file Table_1.DOCX]

Table S1 Biofilm relative inhibition rate of DE17 by 19 kinds of D-AAs

| D-AAs | 0.156mM | 0.313mM | 0.625mM | 1.25mM | 2.5mM | 5mM |
| --- | --- | --- | --- | --- | --- | --- |
| D-Ala | -1.91 | -3.50 | 9.02 | 12.26 | 17.19 | 32.96 |
| D-Val | 3.11 | 1.46 | 3.78 | 4.65 | 0.41 | 15.05 |
| D-Pro | 1.76 | 3.36 | 2.54 | 6.91 | 12.68 | 17.48 |
| D-Leu | 1.15 | 7.13 | 17.18 | 23.88 | 30.60 | 51.01 |
| D-Ile | 6.00 | -0.72 | 4.95 | 4.37 | 5.53 | 6.16 |
| D-Met | 2.01 | 32.74 | 42.31 | 52.39 | 59.07 | 67.35 |
| D-Try | 0.30 | 2.58 | 11.12 | 17.16 | 30.35 | 69.04 |
| D-Phe | 0.23 | 0.11 | 7.90 | 15.17 | 25.28 | 38.86 |
| D-Ser | 0.41 | 8.85 | 7.40 | 24.63 | 36.27 | 45.94 |
| D-Thr | 2.26 | 1.06 | 8.58 | 16.94 | 27.26 | 41.34 |
| D-Cys | -0.58 | 7.41 | 12.63 | 15.78 | 21.25 | 38.75 |
| D-Tyr | 14.96 | 31.92 | 59.09 | 73.95 | 82.19 | 88.64 |
| D-Asp | -3.09 | -0.79 | 5.60 | 13.38 | 22.89 | 30.34 |
| D-Asn | 0.88 | 3.16 | 8.70 | 12.67 | 30.96 | 41.88 |
| D-Glu | 2.44 | 2.94 | 9.99 | 16.41 | 23.11 | 32.03 |
| D-Gln | 0.40 | 0.23 | 0.50 | 9.92 | 30.16 | 47.47 |
| D-Arg | -0.80 | -1.07 | 4.69 | 13.45 | 30.52 | 45.65 |
| D-His | -0.59 | -0.57 | 10.21 | 20.40 | 32.28 | 45.84 |
| D-Lys | 0.79 | 1.92 | 11.34 | 16.63 | 29.76 | 42.64 |
