# Supplementary material for: D-amino acid enhanced the sensitivity of avian pathogenic Escherichia coli to tetracycline and amikacin
Source: Front Vet Sci. 2025 Mar 19;12:1553937. doi: 10.3389/fvets.2025.1553937 (PMC11962725; doi:10.3389/fvets.2025.1553937)
Supplement: Supplementary file 2 [file Table_2.DOCX]

Table S2 Biofilm relative inhibition rate of 20170119 by 19 kinds of D-AAs

| D-AAs | 0.156mM | 0.313mM | 0.625mM | 1.25mM | 2.5mM | 5mM |
| --- | --- | --- | --- | --- | --- | --- |
| D-Ala | -1.31 | -0.62 | 6.72 | 12.27 | 18.35 | 32.45 |
| D-Val | 5.63 | 1.52 | 4.47 | -0.32 | 7.01 | 18.37 |
| D-Pro | 6.67 | 4.51 | 5.46 | 9.86 | 14.94 | 19.36 |
| D-Leu | 1.60 | 0.82 | 16.05 | 24.16 | 41.47 | 58.69 |
| D-Ile | 3.16 | 6.37 | 11.56 | 10.49 | 16.27 | 46.41 |
| D-Met | 5.02 | 32.35 | 41.98 | 52.11 | 58.83 | 67.16 |
| D-Try | 1.18 | 3.98 | 10.45 | 17.32 | 47.68 | 69.14 |
| D-Phe | 2.56 | 6.22 | 11.43 | 16.00 | 27.12 | 34.40 |
| D-Ser | 19.88 | 20.29 | 17.52 | 21.94 | 32.17 | 30.06 |
| D-Thr | -5.19 | -1.36 | 3.94 | 13.86 | 16.99 | 29.56 |
| D-Cys | 1.77 | 10.13 | 10.46 | 16.34 | 23.87 | 36.23 |
| D-Tyr | 19.96 | 31.66 | 59.33 | 73.75 | 82.06 | 88.71 |
| D-Asp | 5.17 | 8.35 | 12.05 | 17.85 | 27.86 | 34.13 |
| D-Asn | -1.03 | 1.29 | 6.91 | 6.18 | 5.90 | 13.15 |
| D-Glu | 2.65 | 5.40 | 7.60 | 16.44 | 23.93 | 31.26 |
| D-Gln | 0.81 | -1.09 | 2.17 | 2.72 | 9.75 | 27.69 |
| D-Arg | 1.54 | 1.67 | 3.79 | 15.35 | 32.94 | 44.86 |
| D-His | 0.84 | -0.57 | 10.21 | 18.28 | 18.07 | 32.68 |
| D-Lys | 2.26 | 1.88 | 3.52 | 12.75 | 15.22 | 29.76 |
